# Impact of Immune Checkpoint Inhibitors on Second Primary Cancer Risk in Patients With Metastatic Lung Cancer Using Real-World Data From the TriNetX Network: Retrospective Cohort Study

**DOI:** 10.2196/64900

**Published:** 2025-10-21

**Authors:** Pierre Heudel, Gema Hernandez, Olivier Denquin, Hugo Crochet, Jean-Yves Blay

**Affiliations:** 1 Medical Oncology Department, Centre Léon Bérard, 28 rue LaennecLyon, 69008, France, 33478782952; 2 TriNetX Europe NV, St.Martens-Latem, Belgium

**Keywords:** second primary cancer, lung cancer, immunotherapy, immune checkpoints inhibitors, second malignant neoplasia, impact, cancer risk, metastatic, cancer patients, cohort, utilization, real world data

## Abstract

**Background:**

Survivors of metastatic lung cancer (MLC) face a heightened risk of developing second primary cancers (SPCs), which significantly impact long-term outcomes. Immune checkpoint inhibitors (ICIs) have revolutionized cancer treatment, but their potential role in reducing SPC risk remains underexplored. This study investigates the association between ICI treatment and the incidence of SPCs in a large, real-world cohort of patients with MLC.

**Objective:**

This study aims to evaluate whether treatment with ICIs is associated with a reduced risk of developing SPCs in patients with metastatic or locally advanced lung cancer, using real-world data from the TriNetX global health research network.

**Methods:**

We conducted a retrospective cohort study using the TriNetX Global Collaborative Network, which aggregates deidentified electronic health records from more than 135 million patients. Adults diagnosed with MLC between February 2004 and February 2024 were included. Patients were divided into 2 cohorts based on ICI exposure. Propensity score matching was applied to balance baseline characteristics. Kaplan-Meier survival analysis and Cox proportional hazards models were used to assess the incidence of SPCs and the composite outcome of SPC or death.

**Results:**

Among 2844 eligible patients, 685 received ICIs and 2157 did not. After propensity score matching, both cohorts included 685 patients. The 5-year incidence of SPCs was lower in the ICI group (1.5%) compared to the non-ICI group (4.2%), with a hazard ratio of 0.49 (95% CI 0.24‐1.01), suggesting a potential protective effect. Furthermore, ICI treatment was significantly associated with a reduced risk of the composite outcome of SPC or death (hazard ratio 0.74, 95% CI 0.62‐0.89). Median follow-up was 20.2 (IQR 60-not reached) months for the ICI group and 68.4 (IQR 36-not reached) months for the non-ICI group.

**Conclusions:**

In this large real-world cohort, ICI treatment was associated with a lower risk of developing SPCs and improved overall outcomes in patients with MLC. These findings support the hypothesis that ICIs may offer a preventive benefit beyond their primary oncologic indications. While the retrospective nature and data limitations warrant cautious interpretation, this study underscores the value of real-world evidence in identifying novel therapeutic benefits and guiding future prospective research.

## Introduction

The trajectory of cancer treatment has undergone a transformative evolution, heralding the integration of immune checkpoint inhibitors (ICIs) as a seminal component in the therapeutic armamentarium [1].

This shift has been characterized by innovative strategies encompassing neoadjuvant, adjuvant, and, more recently, perioperative applications of ICIs, changing treatment paradigms and integrating immunotherapies to improve therapeutic outcomes and patient survival [2]. These strategies aspire to enhance the efficacy of primary tumor management, reducing disease relapse, enabling less invasive surgical interventions, and targeting minimal residual diseases in a spectrum of cancer types.

A recently identified positive facet of significant interest is the potential role of ICIs in mitigating the risks associated with the occurrence of second primary cancers (SPCs)—observed in over 7% of cancer survivors [3]. Amidst a confluence of risk factors such as genetics, environmental exposures, and therapeutic side effects, SPCs emerge as a significant risk impacting long-term survival of cured patients, warranting innovative preventive strategies. A retrospection into clinical data suggests a spectrum of impacts associated with ICIs usage, ranging from associations with reduced SPC occurrences [4-7] to a lack of conclusively protective effects [8,9]. This nuanced clinical tableau underscores the inherent complexities and variables, including histologic considerations and therapeutic contexts, that influence the potential effectiveness of ICIs as a preventive arsenal against SPCs. Although a phase II randomized trial is currently enrolling to answer this important question (NCT05855811) [10], it seems fundamental to continue evaluating the potential impact of ICIs on the risk of SPC, using new extensive retrospective cohorts of patients with cancer. That is why, therefore, we analyzed data from the TriNetX global health research platform that includes data on 30 million patients from Europe to the United States to contribute to improving scientific knowledge [11,12].

The primary objective of this study is to use real-world data from the TriNetX federated data network to investigate the impact of ICIs on the risk of SPC in patients treated for metastatic or locally advanced lung cancer. This exploration aims to provide insights into the potential of ICIs to serve as a preventive strategy against SPCs, thereby informing clinical practice and guiding future research in this critical area.

## Methods

### Data Source and Patient Selection

This noninterventional, retrospective cohort study used data from the international TriNetX Global Collaborative research data network. The primary goal of this research is to evaluate the effect of ICIs treatment for metastatic lung cancer (MLC) on the likelihood of developing a SPC outside the lungs in a real-world scenario. We studied patients from TriNetX with MLC aged 18 years or older. TriNetX is a global collaborative research network that provides real-time access to electronic medical records (diagnoses, procedures, medications, laboratory values, and genomic information) from more than 135 million patients from 114 health care organizations (HCOs), primarily from North America and Western Europe [12]. All data collection, processing, and transmission is done in compliance with all Data Protection laws applicable to the contributing HCOs, including the EU Data Protection Law Regulation 2016/679, the General Data Protection Regulation (GDPR) on the protection of natural persons with regard to the processing of personal data, and the Health Insurance Portability and Accountability Act (HIPAA), the US federal law that protects the privacy and security of health care data. The Global Collaborative Network is a distributed network, and analytics are performed at the HCO, with only aggregate results being returned to the platform.  Individual personal data does not leave the HCO. TriNetX is ISO 27001:2013 certified and maintains a robust IT security program that protects both personal data and health care data. Data collection and quality control methods have been described [11]. As a federated network, research studies using TriNetX are compliant with requests from ethics committees of all contributing countries [13]. To comply with legal frameworks and ethical guidelines guarding against data reidentification, the identity of participating HCOs and their individual contribution to each dataset are not disclosed.

Selected patients were men or women, aged 18 or older, with a diagnosis of MLC between February 1, 2004, and February 1, 2024, to capture the full evolution of ICI use in clinical practice. This extended timeframe allows for robust comparisons across different treatment eras and reflects real-world changes in therapeutic strategies over time. SPCs were identified using *International Classification of Diseases* codes for new primary malignancies at anatomical sites other than the lung, with a minimum latency of 6 months after the initial lung cancer diagnosis, to distinguish them from recurrences or metastases [14]. Due to data limitations, most SPCs identified were metachronous, and synchronous cases could not be reliably assessed. Patients were excluded when (1) a patient died or had a non–lung cancer diagnosis within the first 6 months after the metastasis diagnosis, (2) patients never had a visit to the HCO before the metastasis diagnosis, (3) patients never had a follow-up visit after 6 months of the metastasis diagnosis, (4) patients had lung cancer as the SPC given the difficulty in clinical practice in differentiating a second primary lung cancer from a new metastatic localization of the treated lung cancer, and (5) patients did not have any data of treatment after the metastasis. The initial group of participants was then divided into 2 cohorts. The “ICI” group included patients treated with ICI after the metastasis diagnosis and the “non-ICI” group included patients never treated with ICI. For this study, data were collected until February 25, 2024 (ie, database extraction date). The details on the codes and criteria used to define the cohorts can be found in the MultimediaAppendices 1  2.

### Statistical Analysis

All the statistical analysis was done using the TriNetX platform’s built-in analysis features. ICI and non-ICI cohorts were propensity score matched (PSM) before the comparison on age at the moment of the metastasis diagnosis, gender, race (when available), de novo MLC, smoking, and patient dependency status [15,16]. The index event of both cohorts was the metastasis diagnosis, and the follow-up time window was 10 years.

Baseline characteristics before and after PSM are presented with the number and percentage for categorical variables and mean and SD when variables are numeric. Categorical variables are compared between cohorts with the Fisher exact test *P* value and the standardized mean differences. Numeric variables are compared with the *t* test *P* value and standard difference.

Kaplan-Meier analysis was performed on the matched cohorts to calculate the time between the MLC diagnosis and the SPC. Cox proportional hazard ratios (HRs) and 95% CIs are presented.

### Ethical Considerations

This study was conducted using data from the TriNetX Global Collaborative Network, which provides access to aggregated and deidentified electronic health records. As such, it was exempt from institutional ethics review [17]. All data handling complied with relevant data protection regulations, including the GDPR in the European Union and the HIPAA in the United States. Data collection and quality control methods have been described [11], and research studies using the TriNetX federated network are compliant with ethics committee requirements of all contributing countries [13]. To safeguard participant privacy, only aggregated counts and deidentified information are used, and no protected health information or personal data is made available to platform users. The process of deidentification has been formally attested by a qualified expert in accordance with Section §164.514(b)(1) of the HIPAA Privacy Rule (last updated in December 2020). As such, additional institutional review board approval or informed consent is not required for studies performed within TriNetX. This formal determination by a qualified expert, refreshed in December 2020, supersedes the need for TriNetX’s previous waiver from the Western Institutional Review Board.

## Results

The current analysis considered the 2844 patients with metastatic primary lung cancer selected in the TriNetX network. The study population included 685 patients in the ICI group and 2,157 in the non-ICI group. Patient characteristics are summarized in Table 1. After PSM, both cohorts included 685 patients. A total of 251 (36.6 %) in ICI and 252 (36.8%) non-ICI patients were women, with a mean age of 66.8 (SD 9.7) years and 67 (SD 10) years, respectively. Among them, 216 (31.5%) patients had de novo metastatic disease and 171 (25%) were smokers or former smokers in the ICI cohort. In addition to ICI, 684 of 685 (99.9%) patients in the exposure cohort received at least 1 chemotherapy regimen, and 75 of 685 (10.9%) patients received at least 1 more targeted treatment. Concerning locoregional treatment, 231 of 685 (33.7%) patients were treated by radiotherapy and 41 of 685 (6%) patients had surgery in a metastatic setting. The median follow-up was 20.2 (IQR 60-not reached) months for ICI and 68.4 (IQR 36-not reached) months for non-ICI.

**Table 1.** Characteristics of the population before and after propensity score matching

		Characteristics before propensity score matching			Characteristics after propensity score matching		
		ICI^a^ cohort (n=687)	Non-ICI cohort (n=2157)	*P* value	ICI cohort (n=685)	Non-ICI cohort (n=685)	*P* value
Demographics							
Age (years), median (IQR)		66 (58‐73)	68 (60‐76)	<.001	66 (58‐73)	66 (58‐73)	.64
Female, n (%)		251 (36.5)	868 (40.2)	.11	251 (36.6)	252 (36.8)	.94
Charlson Comorbidity Index, median (IQR)		2 (1‐4)	3 (2‐5)	<.001	2 (1‐4)	2 (1‐4)	.58
Autoimmune disease, n (%)		36 (5.2)	132 (6.1)	.46	36 (5.3)	32 (4.7)	.64
Previous organ transplant, n (%)		5 (0.7)	41 (1.9)	.04	5 (0.7)	4 (0.6)	.75
Diagnosis, n (%)							
Cancer type							
Lung		251 (36.5)	643 (29.8)	.002	250 (36.5)	248 (36.2)	.91
Melanoma		110 (16)	271 (12.6)	.03	110 (16.1)	115 (16.8)	.72
Kidney		61 (8.9)	177 (8.2)	.64	61 (8.9)	60 (8.8)	.95
Treatment, n (%)							
Prior chemotherapy		687 (100)	1785 (82,4)	<.001	684 (99.9)	562 (82)	<.001
Prior radiotherapy		231 (33.7)	580 (26.9)	.13	207 (30.2)	215 (31.4)	.67
Prior surgery		41 (6)	173 (8)	.94	41 (6)	31 (5.6)	>.99

a ICI: immune checkpoint inhibitor.

**Figure 1.**
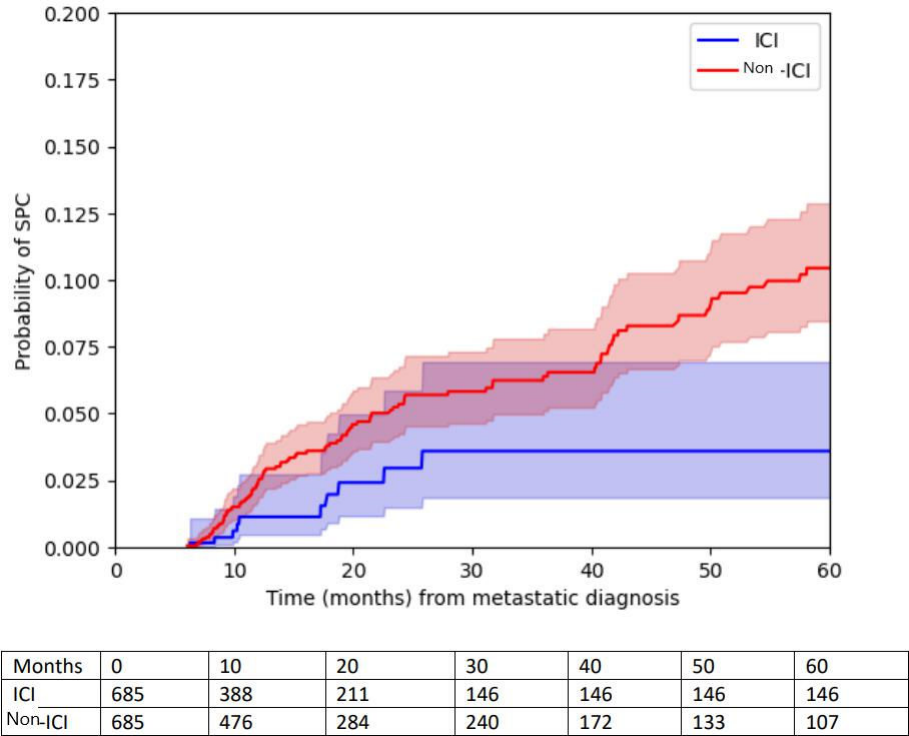
Kaplan-Meier curve for second primary cancer. ICI: immune checkpoint inhibitor; SPC: second primary cancer.

Overall, 39 of 685 (2.8%) patients had a diagnosis of SPC, out of which, 10/685 (1.5%) patients with SPC were reported in the cohort of receiving ICI for their lung cancer in a median interval of 13.9 months (IQR 7-22) versus 23 (3.6%) in a median interval of 17.8 (IQR 9-28) months in patients who did not receive ICI (HR 0.49, 95% CI 0.24‐1.01; *P*=.05) (Figure 1). Among the patients who received ICI, the median time between the diagnosis of advanced or metastatic LC and ICI was 34 days (IQR 21-68 days). The median time between ICI initiation and the diagnosis of SPC was 9 (IQR 1.4‐15.1) months.

Age, gender, race, de novo MLC, smoking, and patient dependency status were identified as potential confounding variables and were included in the propensity score analyses. Standardized mean differences after weighing were within ±0.1 for all observed confounding variables (Multimedia Appendix 1).

**Figure 2.**
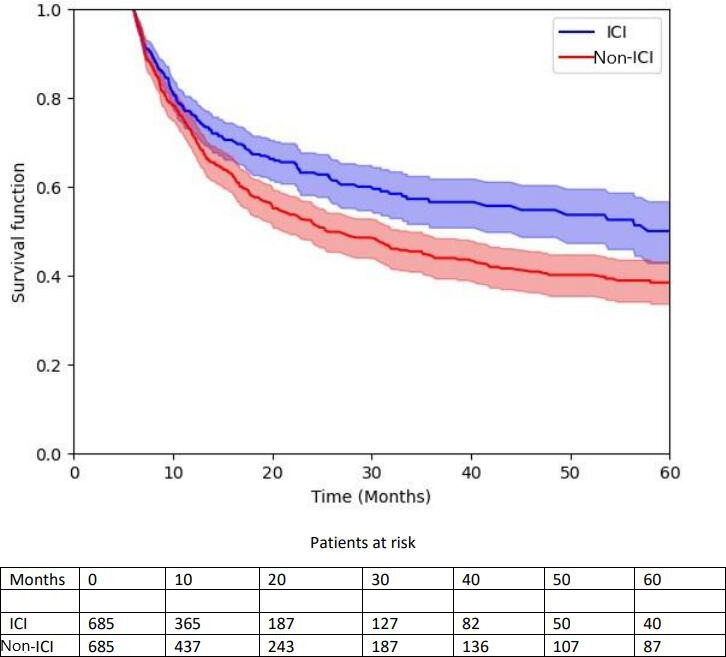
Kaplan-Meier survival curve to death or second primary cancer. ICI: immune checkpoint inhibitor.

Figure 2 presents the time from the diagnosis of MLC to the date of SPC diagnosis or the date of death in patients having received ICI or not for lung cancer. Treatment with ICI for lung cancer was associated with a significant risk reduction of SPC or death (HR 0.74, 95% CI 0.62‐0.89; *P*<.01). The median months to MLC or death were 9.6 (IQR 4-20)and 11.2 (IQR 5-25) for patients treated with ICI and not treated with ICI, respectively.

## Discussion

### Principal Findings

Our study, encompassing 2844 patients with metastatic primary lung cancer from the TriNetX network, indicates a significant impact of ICIs on the incidence of secondary primary cancers and overall survival, showcasing the power of large-scale data in generating valuable insights. This study highlights the critical role of big data and digital health in advancing cancer treatment and research. The TriNetX network, encompassing numerous international centers, exemplifies how big data can be leveraged to conduct extensive and robust studies. The analysis, balanced post-PSM with 685 patients in each cohort, revealed that ICI treatment was associated with a reduced risk of developing SPCs. Specifically, the incidence of SPCs excluding lung cancer was 1.5% in the ICI group compared to 3.6% in the non-ICI group, translating into a risk difference of −0.028 and a favorable HR of 0.49. In addition, our findings demonstrated a reduction in the risk of SPC or death in patients treated with ICIs, with a significant HR of 0.78. This suggests not only the efficacy of ICIs in treating primary tumors but also their potential role in preventing secondary malignancies. The median time to MLC or death was notably higher in the ICI-treated group, further emphasizing the survival benefits of ICIs. The interpretation of the composite endpoint “SPC or death” is limited by the potential confounding effect of overall survival, as death may censor the observation of SPCs. Moreover, patients treated with ICIs may live longer, thereby increasing their window of risk for developing SPCs, which could paradoxically elevate SPC incidence in this group despite a protective effect of immunotherapy.

### Comparison With Prior Work

Our findings align with prior studies suggesting a decreased incidence of patients with SPCs treated with ICIs, such as those by Heudel et al [4-7]. However, our work extends these observations by analyzing a larger, more recent, and real-world cohort of patients with MLC (N=2844) with PSM and an updated follow-up through 2024. Unlike previous studies that included various primary tumor types, we focused specifically on metastatic or locally advanced lung cancer, enhancing the interpretability within a defined clinical context. Moreover, our study leverages the robust infrastructure of the TriNetX platform [18], enabling federated access to real-world data across multiple international HCOs and offering methodological transparency and reproducibility. Recent immunogenetic research supports a biological rationale for ICI-mediated protection: a large UK Biobank study identified specific HLA alleles associated with either increased or decreased SPC risk, suggesting that the adaptive immune system, and thus immune modulation via ICIs, may play a key role in SPC prevention [18]. In addition, population-level evidence from Sweden highlighted strong bi-directional associations between myeloid malignancies and various SPCs, likely involving immune dysfunction or treatment-induced changes [19]. Taken together, these studies reinforce the hypothesis that immune surveillance mechanisms are central to SPC susceptibility. Our results, combined with this growing body of evidence, support the concept that ICIs may offer not only survival benefits but also a protective effect against SPCs, especially in patients with immune-related predispositions.

### Strengths and Limitations

Of course, our study of many limitations began with its retrospective nature. Even if the TriNetX network is extremely important, with numerous international centers, it clearly appears that the scope of the available data remains limited since we do not have, for example, the histological subtypes, or the smoking status, and even less the molecular characteristics of cancers. These missing data constitute a major limitation to the conclusions of our study and to the question of the accessibility and the sharing of these data, which are necessarily present in the electronic medical records [20]. Learned societies have recently established recommendations on real-world data studies, the quality of the conclusions of which is directly linked to the quality of the data obtained [21].

However, the effectiveness of big data and digital health hinges on the quality and comprehensiveness of the data. We must not forget the importance of real-world data that are complementary to clinical trials [22], particularly on subjects such as the appearance of a SPC for which these patients are often excluded from traditional clinical research studies. It therefore appears fundamental to continue collaboration within, for example, the TriNetX network because the greater the volume and completeness of the data, the greater the confidence in the conclusions of these retrospective studies [12]. For this, it seems necessary for each hospital to define its digital data strategy, which must include a data collection strategy (by aligning them from the start with international benchmarks), for the qualification of this data, for their analysis with quality control, and a sharing strategy [23]. This entire strategy must, of course, respect the regulations in force and integrate a priori the question of interoperability to facilitate data sharing and enhance research outcomes [24,25].

### Future Directions

To maximize the potential of big data and digital health, continued collaboration within networks such as TriNetX is essential. The greater the volume and completeness of the data, the higher the confidence in the study conclusions. Recommendations from learned societies on real-world data studies emphasize the importance of data quality, suggesting that the robustness of study conclusions is directly linked to the quality of the data obtained.

### Conclusions

In conclusion, our study showed that patients treated with ICIs for a first lung cancer had a reduced risk of an SPC and underscores the significant benefits of using big data and digital health in cancer research. The ability to conduct real-life studies on an international network such as TriNetX allows for the inclusion of diverse patient populations and the generation of real-world evidence that complements clinical trials. Despite current data limitations, continued network collaborations and strategic data management can enhance the relevance and impact of real-world data studies, ultimately improving patient outcomes and advancing medical knowledge.

## Supplementary material

10.2196/64900 Multimedia Appendix 1 Definition of immune checkpoint inhibitor cohort.

10.2196/64900 Multimedia Appendix 2 Definition of nonimmune checkpoint inhibitor cohort.

10.2196/64900 Checklist 1 RECORD checklist.

## Abbreviations:

GDPR: General Data Protection Regulation
HCO: health care organization
HIPAA: Health Insurance Portability and Accountability Act
HR: hazard ratio
ICI: immune checkpoint inhibitor
MLC: metastatic lung cancer
PSM: propensity score matching
SPC: second primary cancer
