# Supplementary material for: Impact of Immune Checkpoint Inhibitors on Second Primary Cancer Risk in Patients With Metastatic Lung Cancer Using Real-World Data From the TriNetX Network: Retrospective Cohort Study
Source: JMIR Cancer. 2025 Oct 21;11:e64900. doi: 10.2196/64900 (PMC12539796; doi:10.2196/64900)
Supplement: Multimedia Appendix 1 [file cancer-v11-e64900-s001.docx]

**Table 1**.

|  | | | | | |
| --- | --- | --- | --- | --- | --- |
| Group 1 | | | | | |
|  | **Group 1A LC** | | | | |
|  | must have |  | diagnosis | UMLS:ICD10CM:C34 | Malignant neoplasm of bronchus and lung |
|  | date constraint | | This group occurred since 20 years ago | | |
|  | event relationship | | The first instance of Metastasis occurred on or after the first instance of LC | | |
|  | **Group 1B Metastasis** | | | | |
|  | must have | any of | diagnosis | UMLS:ICD10CM:C77 | Secondary and unspecified malignant neoplasm of lymph nodes |
|  |  |  | diagnosis | UMLS:ICD10CM:C78 | Secondary malignant neoplasm of respiratory and digestive organs |
|  |  |  | diagnosis | UMLS:ICD10CM:C79 | Secondary malignant neoplasm of other and unspecified sites |
| Group 2 | | | | | |
|  | **Group 2A Metastasis** | | | | |
|  | must have | any of | diagnosis | UMLS:ICD10CM:C77 | Secondary and unspecified malignant neoplasm of lymph nodes |
|  |  |  | diagnosis | UMLS:ICD10CM:C78 | Secondary malignant neoplasm of respiratory and digestive organs |
|  |  |  | diagnosis | UMLS:ICD10CM:C79 | Secondary malignant neoplasm of other and unspecified sites |
|  | date constraint | | The terms in this group occurred at any time | | |
|  | event relationship | | Any instance of Death or non-LC occurred before or up to 6 months after the first instance of Metastasis | | |
|  | **Group 2B Death or non-LC** | | | | |
|  | cannot have |  | demographics | Deceased | Deceased |
|  |  | or | diagnosis | UMLS:ICD10CM:R99 | Ill-defined and unknown cause of mortality |
|  |  | or | diagnosis | UMLS:ICD10CM:C00-C14 | Malignant neoplasms of lip, oral cavity and pharynx |
|  |  | or | diagnosis | UMLS:ICD10CM:C15-C26 | Malignant neoplasms of digestive organs |
|  |  | or | diagnosis | UMLS:ICD10CM:C40-C41 | Malignant neoplasms of bone and articular cartilage |
|  |  | or | diagnosis | UMLS:ICD10CM:C43-C44 | Melanoma and other malignant neoplasms of skin |
|  |  | or | diagnosis | UMLS:ICD10CM:C45-C49 | Malignant neoplasms of mesothelial and soft tissue |
|  |  | or | diagnosis | UMLS:ICD10CM:C50-C50 | Malignant neoplasms of breast (C50) |
|  |  | or | diagnosis | UMLS:ICD10CM:C51-C58 | Malignant neoplasms of female genital organs |
|  |  | or | diagnosis | UMLS:ICD10CM:C60-C63 | Malignant neoplasms of male genital organs |
|  |  | or | diagnosis | UMLS:ICD10CM:C64-C68 | Malignant neoplasms of urinary tract |
|  |  | or | diagnosis | UMLS:ICD10CM:C69-C72 | Malignant neoplasms of eye, brain and other parts of central nervous system |
|  |  | or | diagnosis | UMLS:ICD10CM:C73-C75 | Malignant neoplasms of thyroid and other endocrine glands |
|  |  | or | diagnosis | UMLS:ICD10CM:C30 | Malignant neoplasm of nasal cavity and middle ear |
|  |  | or | diagnosis | UMLS:ICD10CM:C31 | Malignant neoplasm of accessory sinuses |
|  |  | or | diagnosis | UMLS:ICD10CM:C32 | Malignant neoplasm of larynx |
|  |  | or | diagnosis | UMLS:ICD10CM:C33 | Malignant neoplasm of trachea |
|  |  | or | diagnosis | UMLS:ICD10CM:C37 | Malignant neoplasm of thymus |
|  |  | or | diagnosis | UMLS:ICD10CM:C38 | Malignant neoplasm of heart, mediastinum and pleura |
|  |  | or | diagnosis | UMLS:ICD10CM:C39 | Malignant neoplasm of other and ill-defined sites in the respiratory system and intrathoracic organs |
|  |  | or | diagnosis | UMLS:ICD10CM:C81-C96 | Malignant neoplasms of lymphoid, hematopoietic and related tissue |
| Group 3 | | | | | |
|  | **Group 3A Metastasis** | | | | |
|  | must have | any of | diagnosis | UMLS:ICD10CM:C77 | Secondary and unspecified malignant neoplasm of lymph nodes |
|  |  |  | diagnosis | UMLS:ICD10CM:C78 | Secondary malignant neoplasm of respiratory and digestive organs |
|  |  |  | diagnosis | UMLS:ICD10CM:C79 | Secondary malignant neoplasm of other and unspecified sites |
|  | date constraint | | The terms in this group occurred at any time | | |
|  | event relationship | | Any instance of Baseline visit occurred at least 1 day before the first instance of Metastasis | | |
|  | **Group 3B Baseline visit** | | | | |
|  | must have |  | visit | TNX:Visit | Visit |
| Group 4 | | | | | |
|  | **Group 4A Metastasis** | | | | |
|  | must have | any of | diagnosis | UMLS:ICD10CM:C77 | Secondary and unspecified malignant neoplasm of lymph nodes |
|  |  |  | diagnosis | UMLS:ICD10CM:C78 | Secondary malignant neoplasm of respiratory and digestive organs |
|  |  |  | diagnosis | UMLS:ICD10CM:C79 | Secondary malignant neoplasm of other and unspecified sites |
|  | date constraint | | The terms in this group occurred at any time | | |
|  | event relationship | | Any instance of Follow up visit occurred at least 6 months after the first instance of Metastasis | | |
|  | **Group 4B Follow up visit** | | | | |
|  | must have |  | visit | TNX:Visit | Visit |
| Group 5 | | | | | |
|  | **Group 5A Metastasis** | | | | |
|  | must have | any of | diagnosis | UMLS:ICD10CM:C77 | Secondary and unspecified malignant neoplasm of lymph nodes |
|  |  |  | diagnosis | UMLS:ICD10CM:C78 | Secondary malignant neoplasm of respiratory and digestive organs |
|  |  |  | diagnosis | UMLS:ICD10CM:C79 | Secondary malignant neoplasm of other and unspecified sites |
|  | date constraint | | The terms in this group occurred at any time | | |
|  | event relationship | | Any instance of ICI occurred at least 1 day after the first instance of Metastasis | | |
|  | **Group 5B ICI** | | | | |
|  | must have | any of | medication | NLM:ATC:L01FF | PD-1/PDL-1 (Programmed cell death protein 1/death ligand 1) inhibitors |
|  |  |  | procedure | UMLS:HCPCS:J9271 | Injection, pembrolizumab, 1 mg |
|  |  |  | procedure | UMLS:HCPCS:J9299 | Injection, nivolumab, 1 mg |
|  |  |  | procedure | UMLS:HCPCS:J9022 | Injection, atezolizumab, 10 mg |
|  |  |  | procedure | UMLS:HCPCS:J9023 | Injection, avelumab, 10 mg |
|  |  |  | procedure | UMLS:HCPCS:J9173 | Injection, durvalumab, 10 mg |
|  |  |  | procedure | UMLS:HCPCS:J9119 | Injection, cemiplimab-rwlc, 1 mg |
|  |  |  | procedure | UMLS:HCPCS:J9272 | Injection, dostarlimab-gxly, 10 mg |
| Group 6 | | | | | |
|  | **Group 6A ICI** | | | | |
|  | must have | any of | medication | NLM:ATC:L01FF | PD-1/PDL-1 (Programmed cell death protein 1/death ligand 1) inhibitors |
|  |  |  | procedure | UMLS:HCPCS:J9271 | Injection, pembrolizumab, 1 mg |
|  |  |  | procedure | UMLS:HCPCS:J9299 | Injection, nivolumab, 1 mg |
|  |  |  | procedure | UMLS:HCPCS:J9022 | Injection, atezolizumab, 10 mg |
|  |  |  | procedure | UMLS:HCPCS:J9023 | Injection, avelumab, 10 mg |
|  |  |  | procedure | UMLS:HCPCS:J9173 | Injection, durvalumab, 10 mg |
|  |  |  | procedure | UMLS:HCPCS:J9119 | Injection, cemiplimab-rwlc, 1 mg |
|  |  |  | procedure | UMLS:HCPCS:J9272 | Injection, dostarlimab-gxly, 10 mg |
|  | date constraint | | The terms in this group occurred at any time | | |
|  | event relationship | | Any instance of Death or non-LC occurred on or before the first instance of ICI | | |
|  | **Group 6B Death or non-LC** | | | | |
|  | cannot have |  | demographics | Deceased | Deceased |
|  |  | or | diagnosis | UMLS:ICD10CM:R99 | Ill-defined and unknown cause of mortality |
|  |  | or | diagnosis | UMLS:ICD10CM:C00-C14 | Malignant neoplasms of lip, oral cavity and pharynx |
|  |  | or | diagnosis | UMLS:ICD10CM:C15-C26 | Malignant neoplasms of digestive organs |
|  |  | or | diagnosis | UMLS:ICD10CM:C40-C41 | Malignant neoplasms of bone and articular cartilage |
|  |  | or | diagnosis | UMLS:ICD10CM:C43-C44 | Melanoma and other malignant neoplasms of skin |
|  |  | or | diagnosis | UMLS:ICD10CM:C45-C49 | Malignant neoplasms of mesothelial and soft tissue |
|  |  | or | diagnosis | UMLS:ICD10CM:C50-C50 | Malignant neoplasms of breast (C50) |
|  |  | or | diagnosis | UMLS:ICD10CM:C51-C58 | Malignant neoplasms of female genital organs |
|  |  | or | diagnosis | UMLS:ICD10CM:C60-C63 | Malignant neoplasms of male genital organs |
|  |  | or | diagnosis | UMLS:ICD10CM:C64-C68 | Malignant neoplasms of urinary tract |
|  |  | or | diagnosis | UMLS:ICD10CM:C69-C72 | Malignant neoplasms of eye, brain and other parts of central nervous system |
|  |  | or | diagnosis | UMLS:ICD10CM:C73-C75 | Malignant neoplasms of thyroid and other endocrine glands |
|  |  | or | diagnosis | UMLS:ICD10CM:C30 | Malignant neoplasm of nasal cavity and middle ear |
|  |  | or | diagnosis | UMLS:ICD10CM:C31 | Malignant neoplasm of accessory sinuses |
|  |  | or | diagnosis | UMLS:ICD10CM:C32 | Malignant neoplasm of larynx |
|  |  | or | diagnosis | UMLS:ICD10CM:C33 | Malignant neoplasm of trachea |
|  |  | or | diagnosis | UMLS:ICD10CM:C37 | Malignant neoplasm of thymus |
|  |  | or | diagnosis | UMLS:ICD10CM:C38 | Malignant neoplasm of heart, mediastinum and pleura |
|  |  | or | diagnosis | UMLS:ICD10CM:C39 | Malignant neoplasm of other and ill-defined sites in the respiratory system and intrathoracic organs |
|  |  | or | diagnosis | UMLS:ICD10CM:C81-C96 | Malignant neoplasms of lymphoid, hematopoietic and related tissue |
| Group 7 | | | | | |
|  | **Group 7A Metastasis** | | | | |
|  | must have | any of | diagnosis | UMLS:ICD10CM:C77 | Secondary and unspecified malignant neoplasm of lymph nodes |
|  |  |  | diagnosis | UMLS:ICD10CM:C78 | Secondary malignant neoplasm of respiratory and digestive organs |
|  |  |  | diagnosis | UMLS:ICD10CM:C79 | Secondary malignant neoplasm of other and unspecified sites |
|  | date constraint | | The terms in this group occurred at any time | | |
|  | event relationship | | Any instance of Second LC diagnosis occurred at least 6 months after the first instance of Metastasis | | |
|  | **Group 7B Second LC diagnosis** | | | | |
|  | cannot have |  | diagnosis | UMLS:ICD10CM:C34 | Malignant neoplasm of bronchus and lung (Data Source: EHR) |
