# Supplementary material for: Impact of Immune Checkpoint Inhibitors on Second Primary Cancer Risk in Patients With Metastatic Lung Cancer Using Real-World Data From the TriNetX Network: Retrospective Cohort Study
Source: JMIR Cancer. 2025 Oct 21;11:e64900. doi: 10.2196/64900 (PMC12539796; doi:10.2196/64900)
Supplement: Checklist 1 [file cancer-v11-e64900-s003.docx]

| Item | Description | Location in Manuscript |
| --- | --- | --- |
| Title and abstract | Indicate the study is observational and describe what was done and found. | Title page, Abstract |
| Background/rationale | Explain the scientific background and rationale for the investigation. | Introduction |
| Objectives | State specific objectives, including any prespecified hypotheses. | Introduction, end of paragraph 2 |
| Study design | Present key elements of study design early in the paper. | Methods - Data source and patient selection |
| Setting | Describe setting, locations, and relevant dates. | Methods - Data source and patient selection |
| Participants | Give eligibility criteria and sources/methods of selection. | Methods - Patient selection |
| Variables | Clearly define all outcomes, exposures, predictors, confounders, and effect modifiers. | Methods - Statistical analysis |
| Data sources/measurement | For each variable, give sources of data and details of assessment methods. | Methods - Data source and patient selection |
| Bias | Describe efforts to address potential sources of bias. | Methods - Propensity score matching |
| Study size | Explain how study size was arrived at. | Methods - Patient selection |
| Quantitative variables | Explain how quantitative variables were handled in the analyses. | Methods - Statistical analysis |
| Statistical methods | Describe all statistical methods, including those used to control for confounding. | Methods - Statistical analysis |
| Participants (Results) | Report numbers of individuals at each stage of study. | Results - Study population |
| Descriptive data | Give characteristics of study participants. | Results - Table 1 |
| Outcome data | Report numbers of outcome events or summary measures. | Results - SPC incidence |
| Main results | Give unadjusted and adjusted estimates and their precision. | Results - Hazard ratios |
| Other analyses | Report other analyses done, e.g., subgroup and sensitivity analyses. | Results - Composite outcome |
| Key results | Summarize key results with reference to study objectives. | Discussion - Principal findings |
| Limitations | Discuss limitations of the study. | Discussion - Strengths and limitations |
| Interpretation | Give a cautious overall interpretation of results. | Discussion - Conclusion |
| Generalisability | Discuss the generalisability of the study results. | Discussion - Final paragraph |
| Funding | Give the source of funding and role of funders. | Post-manuscript sections - Funding statement |
| Ethics | Describe ethics approval and data protection. | Methods - Ethical Considerations |
| Data access and cleaning | Describe data access and cleaning methods. | Methods - TriNetX platform description |
| Linkage | State whether the study included data linkage. | Not applicable - single source (TriNetX) |
